# Energy refinement and analysis of structures in the QM9 database via a highly accurate quantum chemical method

**DOI:** 10.1038/s41597-019-0121-7

**Published:** 2019-07-03

**Authors:** Hyungjun Kim, Ji Young Park, Sunghwan Choi

**Affiliations:** 1 Department of Chemistry, Incheon National University, 119 Academy-ro, Yeonsu-gu, Incheon 22012 Republic of Korea; 2 Department of Chemistry, Korea Advanced Institute of Science and Technology (KAIST), 291 Daehak-ro, Yuseong-gu, Daejeon 34141 Republic of Korea; 3 National Institute of Supercomputing and Network, Korea Institute of Science and Technology Information, Daejeon, 34141 Republic of Korea

**Keywords:** Quantum chemistry, Computational chemistry, Density functional theory

## Abstract

A wide variety of data-driven approaches have been introduced in the field of quantum chemistry. To extend the applicable range and improve the prediction power of those approaches, highly accurate quantum chemical benchmarks that cover extremely large chemical spaces are required. Here, we report ~134 k quantum chemical calculations performed with G4MP2, the fourth generation of the G-n series in which second-order perturbation theory is employed. A single composite method calculation executes several low-level calculations to reproduce the results of high-level *ab initio* calculations with the aim of saving computational costs. Therefore, our database reports the results of the various methods (e.g., density functional theory, Hartree-Fock, Møller–Plesset perturbation theory, and coupled-cluster theory). Additionally, we examined the structure information of both the QM9 and the revised databases via chemical graph analysis. Our database can be applied to refine and improve the quality of data-driven quantum chemical prediction. Furthermore, we reported the raw outputs of all calculations performed in this work for other potential applications.

## Abstract (summary)

**Table:** 

Design Type(s)	chemical structure classification objective • chemical reaction data analysis objective • modeling and simulation objective
Measurement Type(s)	chemical structure analysis
Technology Type(s)	ab initio quantum chemistry computational method
Factor Type(s)	atom
Sample Characteristic(s)

Machine-accessible metadata file describing the reported data (ISA-Tab format)

## Background & Summary

A large number of chemical databases enable new strategies to solve chemical problems that are difficult to address by existing chemical principles^1–9^. In particular, in the field of functional material/drug design^10,11^ and the investigation of reaction pathways^12^, data-driven approaches such as data mining and machine learning techniques open up a new era beyond traditional quantum chemistry approaches. To enable the further development of such promising applications, quantum chemistry databases that cover a wide range of chemical space with a high accuracy comparable to that of experimental observations are desired^13–17^.

For these purposes, many databases containing quantum chemical calculations have been published. Most of the simulations in those databases were performed with density functional theory (DFT) because of its reasonable accuracy/cost ratio. However, the applicability of DFT throughout the entire chemical universe is questioned^18,19^. In addition, covering the chemical universe with databases relying on a single methodology may introduce a bias to predictive models.

Despite tremendous improvements in computing power, constructing a large quantum chemical database by a highly accurate method is still a challenging and time-consuming problem. The computational cost of the simplest *ab initio* calculation, Hartree-Fock (HF), increases with ~*O(N*^*4*^*)*, where N is the number of basis functions. The computational complexity becomes even worse for high-level calculations. Due to this rapid increase in computational cost, the size of existing databases constructed with high-level calculations is too small to cover general chemical applications^14^. For example, the G3/05 test set, which is used for verifying the Gaussian-4 theory using reduced order perturbation theory (G4MP2) method, contains only 236 experimentally obtained enthalpies of formation, 88 ionization potentials, 58 electron affinities, and 8 proton affinities of organic molecules^20,21^. To the authors’ knowledge, the largest database built with a composite method includes 16k isomers of C_7_H_10_O_2,_ which is only approximately 10% of the database employed in this study. These accumulated data were used to predict the electronic correlation energy by using the kernel regression method^22^.

To determine the accurate structure-property relationship via data-driven approaches, systematic and thorough sampling for a large chemical space is essential. The series of Generated Database-n (GDB-n) which include all possible molecules of up to n nonhydrogen atoms were proposed by the enumeration of chemical graphs^23–25^. The database called QM9, which is a subset of GDB-17, contains all molecules (~134 k) consisting of at most 9 nonhydrogen atoms (carbon, nitrogen, oxygen, and fluorine). For the QM9 set, the geometries and thermodynamic/electronic/energetic properties computed by DFT were reported in a previous report^14^. Although this database has served as the reference chemical space for some chemical problems, the QM9 set is subject the following limitations: (1) A single computational condition was employed, which prohibits investigation into the correlation and difference among various computational conditions. (2) The ability of the B3LYP functional to reproduce high-level *ab initio* calculations is not verified. (3) Only postprocessed information is available, which may limit other interesting approaches.

In this work, we reported the QM9-G4MP2 database, which contains the G4MP2 energies of the refined molecular structures in the QM9 set^26^ as well as the energies from all methods invoked by the G4MP2 calculations (e.g., DFT, HF, Møller–Plesset perturbation theory and coupled-cluster single and double excitations with perturbative triple correction methods). All the raw outputs of the G4MP2 calculations are also published to allow the research community to obtain information that we do not address in this work. These data could prompt other applications, for instance, improving the quality of data-driven approaches by feeding high-quality data to existing models as well as designing new architectures for predictive models to learn quantum chemical properties.

## Methods

Composite methods target highly accurate thermochemical properties (deviation from the experiments less than 1 kcal/mol) within manageable time by performing a series of low-level calculations. This approach is based on the fact that extending correlation energy and basis set effects are additive to a certain degree. The G4MP2 method consists of geometry optimization with B3LYP/6-31 G(2df,p) and single point calculations with CCSD(T,FC)/6-31 G(d), MP2(FC)/G3MP2largeXP, RHF/mod-aug-cc-pVTZ and RHF/mod-aug-cc-pVQZ. The philosophy of Gaussian methods and technical details of the G4MP2 method can be found in the work of Curtiss and his coworkers^20,21^.

The geometries of the QM9 set molecules were reoptimized to ensure convergence to a minimum. All calculations were performed with the *Gaussian 16* package (A.03 version), while the results of the QM9 set were computed with *Gaussian 09*. To obtain atomization energies (AEs), G4MP2 calculations for H, C, N, O, and F atoms were performed. Both the charge and spin multiplicities for all systems are the same as those of the QM9 set.

## Data Records

All raw data, python scripts to construct a database and perform analysis addressed in this paper, and the results of postprocessing can be downloaded from Figshare^27^. Other information that is not included in our table can be obtained by parsing the raw outputs named *dsgdb9nsd_*index*.log*, stored in the output folder. Here, the order of the index is the same as that in the QM9. atom_ref folder containing the output of five (C, H, N, O, F) atoms. Note that not all calculations are performed in identical computational environments. Comparison of the elapsed time among calculations performed in different systems does not represent their relative computational costs. The *result.csv* file contains energies computed from different combinations of computational methods and basis sets. It can be generated by running a *parase.py* script in the same folders. The details of the columns in the *result.csv* file are explained in Table 1. The csv file contains energies for only 133858 cases because the energy values for the molecules whose calculations failed to converge are not included. The indices for 27 structures where we could not obtain converged energies are stored in *index_not_converged.txt*. The usage of published scripts and a description of each file in database can be found in the Usage Notes section.

**Table 1:** A description of column keys for the CSV file containing the data set.

index	index of QM9-G4(MP2) which is the same as that of QM9
B3LYP/6-31 g(2df,p)	Total energy of B3LYP/6-31 g(2df,p)
HF/6-31 g(d)	Total energy of HF/6-31 g(d)
MP2/6-31 g(d)	Total energy of MP2/6-31 g(d)
MP3/6-31 g(d)	Total energy of MP3/6-31 g(d)
MP4D/6-31 g(d)	Total energy of MP4D/6-31 g(d)
MP4DQ/6-31 g(d)	Total energy of MP4DQ/6-31 g(d)
MP4SDTQ/6-31 g(d)	Total energy of MP4SDTQ/6-31 g(d)
MP4SDQ/6-31 g(d)	Total energy of MP4SDQ/6-31 g(d)
CCSD/6-31 g(d)	Total energy of CCSD/6-31 g(d)
CCSD(T)/6-31 g(d)	Total energy of CCSD(T)/6-31 g(d)
HF/G3MP2largeXP	Total energy of HF/G3MP2largeXP
MP2/G3MP2largeXP	Total energy of MP2/G3MP2largeXP
HF/mod-aug-cc-pVTZ	Total energy of HF/mod-aug-cc-pVTZ
HF/mod-aug-cc-pVQZ	Total energy of HF/mod-aug-cc-pVQZ
G4MP2	Internal energy at 0 K of G4MP2

## Technical Validation

The geometry optimization procedure of the G4MP2 calculations is performed with the B3LYP/6-31G(2df,p) condition. To validate the consistency and integrity of the molecular structures in the quantum chemical database, we compare the total energy of B3LYP/6-31G(2df,p) from the QM9-G4MP2 and the QM9 sets, denoted as ${{\rm{E}}}_{B3LYP}^{QM9-G4MP2}$ and E^*QM*9^, respectively. Most of the geometry optimization was performed within the first few steps; therefore, this simple energy difference can quantify the subtle changes in the molecular structure. Since the total energy referred as B3LYP/6-31G(2df,p) in the QM9-G4MP2 database does not include zero-point energy, for the comparison, the reference total energy(E^*QM*9^) is derived by subtracting the zero-point energy from the internal energy at 0 K.

**Fig. 1:**
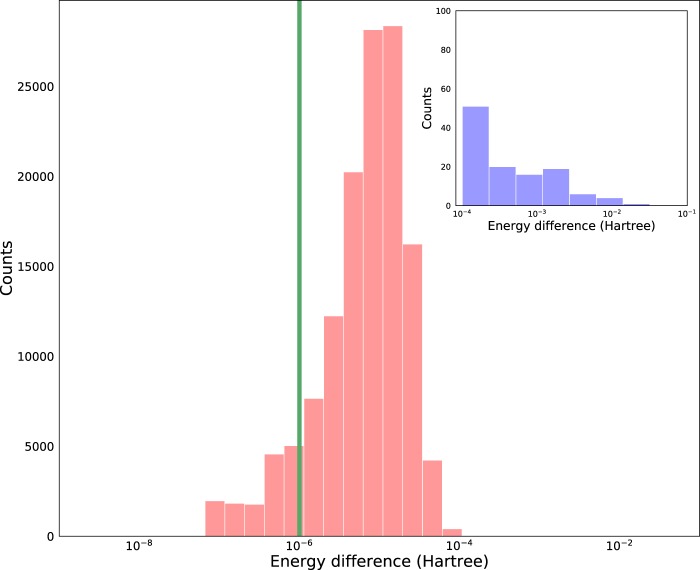
Histogram of the differences in total energy, $| {{\rm{E}}}^{QM9}\,-\,{{\rm{E}}}_{B3LYP}^{QM9-G4MP2}|$. The green vertical line indicates 10^−6^, which is the maximal number of digits that the QM9 set offers. Deviations below 10^−6^ Hartree error are numerically meaningless.

Figure 1 plots the distribution of deviations between E^*QM*9^ and ${{\rm{E}}}_{B3LYP}^{QM9-G4MP2}$. In the QM9 set, both the internal energy and ZPVE are given to six decimal places. Therefore, cases with less than 10^−6^ Hartree differences would be considered the same value. One possible reason our energies have discrepancies with the energies of the QM9 set is the use of different computation options (e.g., opt = diloose and int = ntultrafine) in some cases of the QM9 set to accelerate convergence. Although discordance greater than the numerical tolerance was observed, chemically meaningful differences (>1 kcal/mol) were observed in only a small fraction of molecules (~0.017%).

**Fig. 2:**
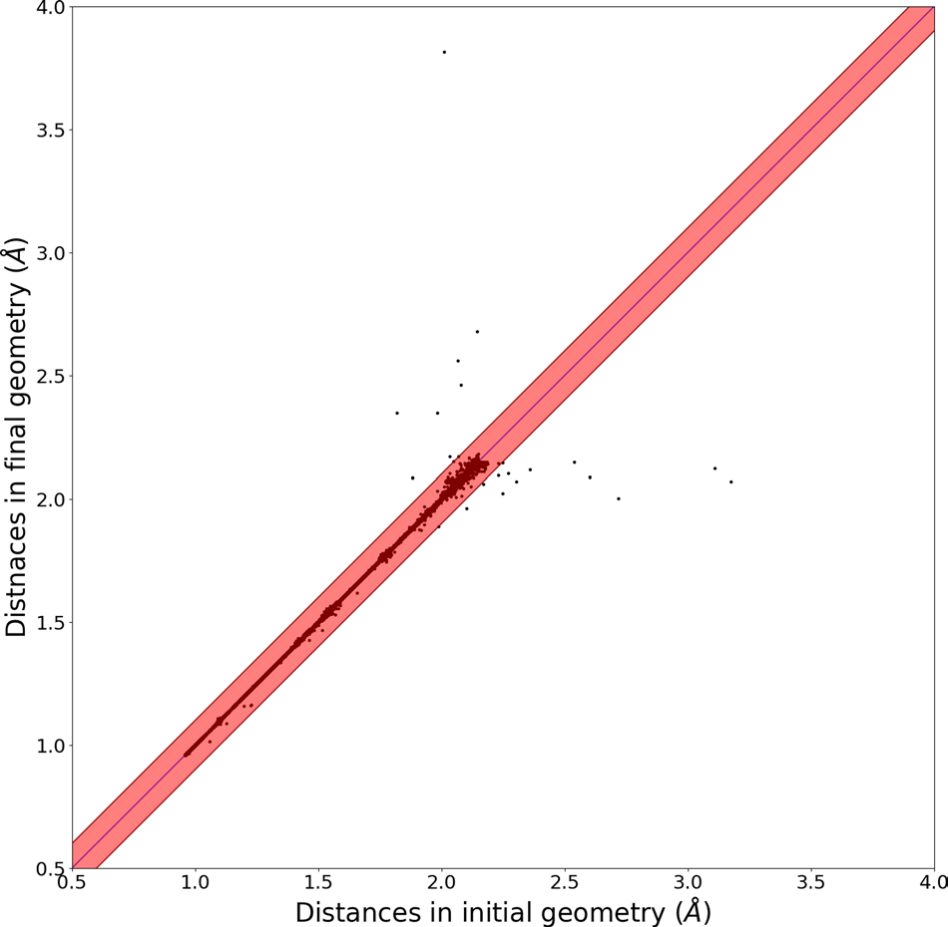
Correlation plot of distances of atomic pairs in the QM9 set (d_QM9_) and the QM9-G4MP2 (d_QM9−G4MP2_).

To quantify the structure difference, we calculate the distances of all atomic pairs across the entire molecular set. For convenience, d_QM9_ and d_QM9–G4MP2_ indicate the sets of distances between all atomic pairs present in the QM9 and the QM9-G4MP2 databases, respectively. Figure 2 depicts the correlation plot between d_QM9_ and d_QM9−G4MP2_ for the cases shorter than 2.15 Å. The specific value of 2.15 Å is the maximum distance at which a chemical bond can be formed between an atomic pair consisting of C, H, O, N, and F atoms. Therefore, Fig. 2 plots the distances of all atomic pairs that are able to form covalent bonds in the QM9 and QM9-G4MP2 structures. The solid black line (y = x line) represents the specific atomic pair where no bond distance change during geometry optimizations is observed. The points above and below the line indicate that the distances are elongated and shortened by the additional optimization, respectively. Most of the points in Fig. 2 are included in the red zone, which indicates a discrepancy of less than 0.1 Å.

**Fig. 3:**
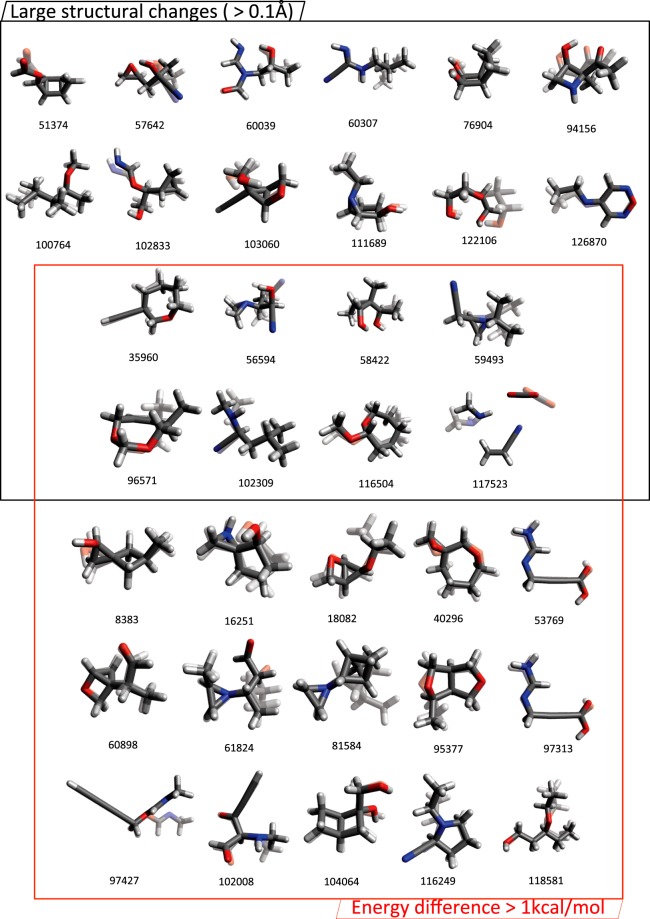
Molecular structures with discrepancies of more than 1 kcal/mol in total energies or more than or 0.1 Å in distances of atomic pairs between the QM9 and QM9-G4MP2 sets. The QM9 and QM9-G4MP2 structures are presented with a 50% transparent and an opaque model, respectively.

Figure 3 summarizes the geometries and indices that undergo large structure/energy changes by further relaxation. These values are divided into two sets, for changes in molecular structure larger than the criteria of 0.1 Å in bond distance in energy (black upper box) and for energy differences (red lower box) larger than the criteria of 1 kcal/mol (=1.59 mHartree).

The 20 geometries in the black box contain atomic pairs with changes larger than 0.1 Å, which correspond to the points outside the red zone in Fig. 2. None of these 20 cases involve bond formation/breaking due to the additional optimization, which means the atomic connectivity is not changed, because the criterion (2.15 Å) is long enough to cover all chemically bonded atomic pairs as well as some nonbonded ones.

For all 23 cases in the red box in Fig. 2, the ${{\rm{E}}}_{B3LYP}^{QM9-G4MP2}$ values are smaller than the corresponding E^*QM*9^ values. This finding means that the newly found conformations in the QM9-G4MP2 set are more stable and likely closer to the global minimum than the QM9 conformations. While the index of 117523 has the largest discrepancy of ~20 kcal/mol, all other cases show 3~5 kcal/mol energy differences. The relatively small energy difference of 3~5 kcal/mol can be sufficiently induced by conformational changes alone, even without connectivity changes. The largest deviation (20 kcal/mol) is observed for the trimolecular systems, which may have many local minima due to complex intermolecular interactions, thus introducing large deviations among conformers.

By definition of the GDB, every structure in the GDB set should be uniquely determined (i.e., there should be no duplicated structures in the GDB set) and should be a single molecule. In Fig. 3, we can find two unexpected types of geometries: 1) duplicated structures and 2) multimolecular systems. The cases 53769 and 97313 contain identical chemical structures, and the case indexed 117523 contains more than one molecule in a single system.

We applied chemical graph representations to both the QM9 and QM9-G4MP2 sets to count the number of duplicate structures and multimolecular systems. Herein, the nodes and edges of graphs represent atoms and chemical bonds. The edges in graphical representations (=chemical bonds) are formed only when the distance of atomic pairs is shorter than the known covalent bond length with a 15% margin^28^. Through isomorphic relations among graphs, we can determine whether chemical structures are the same. Additionally, the number of components of chemical graphs corresponds to the number of molecules in the chemical graph, and it is possible to check the identity of chemical structures.

The indices of 252 duplicated systems in the QM9 and QM9-G4MP2 sets could be identified with the script named *count_duplicate.py*, and its output can be found in *index_duplicated_structure.txt*. Moreover, 229 and 227 systems in the QM9 and QM9-G4MP2 sets, respectively, include more than one molecule. This difference (229 and 227) is caused by the exclusion of the cases indexed 21725 and 87037 from QM9-G4MP2 in the graph analysis due to the abnormal termination of the G4MP2 calculations. We confirmed this analysis by parsing InChI, which distinguishes molecules using dot(.). Therefore, the presence of dot(.) means that the system consists of more than one molecule. The InChI of the QM9 set contains dots for the 229 cases with exactly the same molecules found by *count_duplicate.py*. The indices for these 229 cases are listed in *index_multi_mol.txt*.

These duplicates and multimolecular structures could be caused by the automated structure generation procedure of the QM9 set. Because the initial geometries for QM9-G4MP2 geometry optimization are adopted from the QM9 set, the two types of unwanted cases mentioned above are also observed in the QM9-G4MP2 set. Although the QM9 and QM9-G4MP2 sets do not completely cover the structure of the GDB set due to inappropriate structure optimizations, the number of problematic structures is only a tiny fraction (~0.01%) of the total size (~134 k) of the database. Therefore, both databases covering large samples of small organic molecules are still valid and irreplaceable.

**Fig. 4:**
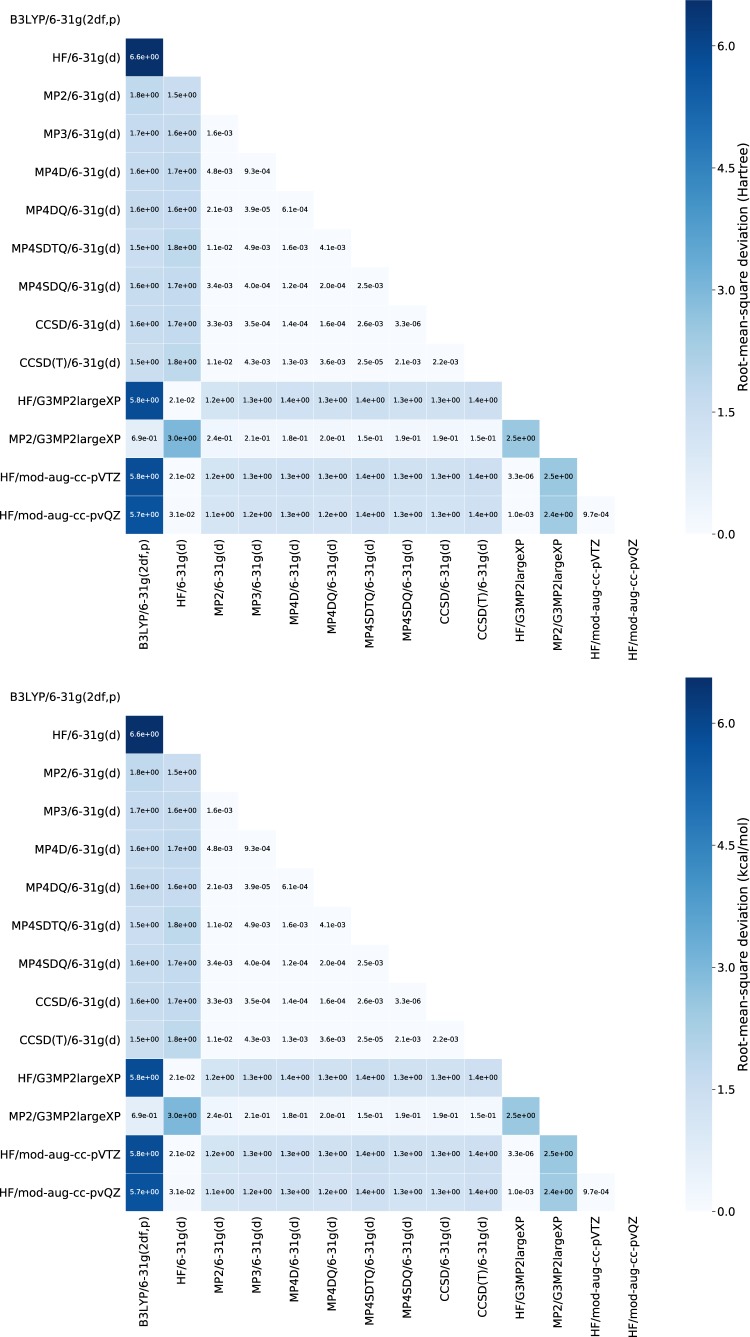
Heatmaps of the root-mean-square deviation between different computational conditions for total electronic energies (in Hartree, top) and atomization energies (in kcal/mol, bottom) of the QM9-G4MP2 sets. The labels of each axis represent the computational conditions invoked by the G4MP2 calculations. The order of computational conditions is given in the order printed in the raw outputs of the G4MP2 calculations.

Figure 4 represents the root-mean-square deviation (RMSD) of total energies and the corresponding AEs among computational methods invoked by G4MP2 calculations. The discrepancies among AEs are relatively smaller than those among the total energies, which means that differences arising from computational methods are decreased by the process of stoichiometric calculations. Thus, many other analyses on the QM9-G4MP2 database can be performed by parsing the outputs of *Gaussian 16*.

## Usage Notes

The QM9-G4MP2 database is provided in a compressed file format. The *data_publication_main.tar.gz* file contains all scripts for validation, atomic results and index files (*index_not_converged.txt*, *index_duplicated_structure.txt*, and *index_multi_mol.txt*). Files starting with ‘output’ contain log files of G4MP2 calculations for molecules. To run scripts with the default options, all outputs whose name ends with log should be positioned in data_publication/output folder, and for the scripts that perform comparisons to the QM9 database, the xyz files from the QM9 database should be placed in data_publication/ref/dsgdb9nsd/. All scripts require the high-level mathematical library Numpy. Some scripts require the QM9 database, which can be downloaded from figshare^26^. The README.md file stored in the same directory contains additional information for each script.

*parse.py*: This script parses all G4MP2 outputs in a directory and generates a CSV file that stores a series of energy values obtained from the lines starting with “\1\1”, which are printed at the end of each computation. All energy values of molecules whose calculations do not end normally are not included, and their indices are printed out during running of the script. Pandas is required to run it. It runs in 2~3 hours on an ordinary personal computer.

*compare_geom.py*: This script calculates and stores the distances between all atomic pairs in the geometries of both the QM9 set and our database. The output of this script can be loaded by pickle.load in python. The indices for distances are presented in the lexicographic sort order by the atomic indices.

*compare_energy.py*: This script extracts the energies of the QM9 set and calculates ${{\rm{E}}}^{QM9}\,-\,{{\rm{E}}}_{B3LYP}^{QM9-G4MP2}$. The output energies are stored in Hartree units. Using numpy.loadtxt(), the output file can be loaded.

*calculate_atomization.py*: This script calculates AEs from ${{\rm{E}}}_{B3LYP}^{QM9-G4MP2}$. To run this script, calculation results for atomic systems are required. Therefore, *parse.py* in the atom_ref folder should be executed before this script.

*count_mol.py*: This script counts molecules in the structures belonging to QM9 and QM9-G4MP2 sets and prints the indices of systems including multiple molecules. A library for the analysis of graphs, NetworkX, is required to run it.

*count_duplicates.py*: This script counts duplicated structures in QM9 and QM9-G4MP2 sets. It takes ~2 days on an ordinary personal computer. A library for analysis of graphs, named NetworkX, is required to run it.

Explanations for all the options of each script can be checked through–help option.

## ISA-Tab metadata file

Download metadata file

## Data Availability

The QM9-G4MP2 database contains raw outputs and scripts to parse the data addressed in this paper. All scripts are released with the BSD license. Other details on the scripts are discussed in the Usage Notes.
